# Survival prediction in patients with colon adenocarcinoma via multiomics data integration using a deep learning algorithm

**DOI:** 10.1042/BSR20201482

**Published:** 2020-12-21

**Authors:** Jiudi Lv, Junjie Wang, Xiujuan Shang, Fangfang Liu, Shixun Guo

**Affiliations:** 1 Department of General Surgery Three, Xinxiang Central Hospital, No. 56 Jinsui Avenue, Xinxiang, Henan 453000, China; 2 Department of Oncology Medicine Three, Xinxiang Central Hospital, No. 56 Jinsui Avenue, Xinxiang, Henan 453000, China; 3 Severe Medical Section, Xinxiang Central Hospital, No. 56 Jinsui Avenue, Xinxiang, Henan 453000, China

**Keywords:** deep learning, methylation, miRNA, multi-omics data

## Abstract

The present study proposed a deep learning (DL) algorithm to predict survival in patients with colon adenocarcinoma (COAD) based on multiomics integration. The survival-sensitive model was constructed using an autoencoder for DL implementation based on The Cancer Genome Atlas (TCGA) data of patients with COAD. The autoencoder framework was compared with PCA, NMF, t-SNE, and univariable Cox-PH model for identifying survival-related features. The prognostic robustness of the inferred survival risk groups was validated using three independent confirmation cohorts. Differential expression analysis, Pearson’s correlation analysis, construction of miRNA–target gene network, and function enrichment analysis were performed. Two risk groups with significant survival differences were identified in TCGA set using the autoencoder-based model (log-rank *P*-value = 5.51e^−07^). The autoencoder framework showed superior performance compared with PCA, NMF, t-SNE, and the univariable Cox-PH model based on the C-index, log-rank *P*-value, and Brier score. The robustness of the classification model was successfully verified in three independent validation sets. There were 1271 differentially expressed genes, 10 differentially expressed miRNAs, and 12 hypermethylated genes between the survival risk groups. Among these, miR-133b and its target genes (*GNB4, PTPRZ1, RUNX1T1, EPHA7, GPM6A, BICC1*, and *ADAMTS5*) were used to construct a network. These genes were significantly enriched in ECM–receptor interaction, focal adhesion, PI3K–Akt signaling pathway, and glucose metabolism-related pathways. The risk subgroups obtained through a multiomics data integration pipeline using the DL algorithm had good robustness. miR-133b and its target genes could be potential diagnostic markers. The results would assist in elucidating the possible pathogenesis of COAD.

## Introduction

Colorectal cancer (CRC) is the fourth most prevalent cancer and the second primary cause of cancer-related death in the United States. [1]. Due to improvements in cancer prevention, screening-based diagnosis, treatment modalities, and other factors, the incidence and mortality rate of CRC have significantly decreased [2]. Nonetheless, prognosis remains poor for patients with advanced colon cancer [3], and 90% of these patients have colon adenocarcinoma (COAD) [4]. Therefore, it is of great practical significance to improve the prognosis of patients with COAD by effective prognostic stratification.

Multiomics data integration provides more information on tumorigenesis and development than 1D omics data and delivers additional benefits for precision medicine [5]. Deep learning (DL) allows the processing of high-dimensional data with numerous features and can use its activation function to utilize complicated nonlinear patterns [6]. An autoencoder, a DL algorithm, can reconstruct original input data to produce new features to represent the dataset. The application of DL algorithms is in its infancy for developing prognostic models. For instance, DL-based multiomics integration is robust in predicting the survival of patients with hepatocellular carcinoma [7]. Moreover, autoencoder-based multiomics integration has been used to identify survival-specific subtypes in patients with high-risk neuroblastoma [8] and bladder cancer [9]. However, further studies are needed to predict the survival rate of patients with COAD by integrating multiomics data through DL.

In the present study, we applied a DL computational framework based on multiomics data (mRNA data, miRNA data, CpG methylation data, and clinical information) from The Cancer Genome Atlas (TCGA) and built a prognostic model based on new features transformed by an autoencoder to stratify patients with COAD. The stratification identified two survival subgroups with significantly different survival rates, which were further successfully validated in three independent datasets. Functional analysis of the two survival subgroups uncovered critical miRNAs, target genes, and signaling pathways in the biology of COAD. The robust classification of patients with COAD using this model may be beneficial for prognosis prediction and the development of precision medicine.

## Methods

### Datasets and preprocessing

We obtained paired RNA sequencing (RNA-seq) data (RNA-seq Illumina HiSeq platform), miRNA sequencing (miRNA-seq) data (miRNA-seq IlluminaHiseq platform), and DNA methylation data (Methylation Illumina 450k platform) of 288 COAD samples with corresponding clinical information from TCGA database as the training set. For preprocessing of raw data, we first removed the probes or genes with values missing in more than 50% of samples. Methylation data were annotated using the RIlluminaHumanMethylation 450kanno.ilmn12.hg19 package [10]. Beta values of several DNA methylation sites in the promoter region were averaged to be the mean promoter methylation value. The samples were deleted if more than 20% of the features were missing. The missing values were filled out using the *impute* package (https://www.bioconductor.org/packages/release/bioc/html/impute.html) of R. Eventually, input features with zero values across all samples were removed. E-GEOD-17538 with 232 samples (A-AFFY-44, RNA-seq), E-GEOD-39582 with 558 samples (A-AFFY-44, RNA-seq), and E-GEOD-28722 with 125 samples (A-GEOD-13425, RNA-seq) were downloaded from the ArrayExpress database (https://www.ebi.ac.uk/arrayexpress/). The three datasets were used as validation sets. Clinical characteristics of TCGA set and the three validation sets are summarized in Table 1. Furthermore, the detailed clinical information of samples in TCGA, E-GEOD-17538, E-GEOD-39582, and E-GEOD-28722 datasets, is shown in Supplementary Tables S1–S4, respectively.

**Table 1:** Clinical features of patients in TCGA dataset and three confirmation cohorts

Variable	TCGA set (*N*=288)	E-GEOD-17538 (*N*=232)	E-GEOD-39582 (*N*=558)	E-GEOD-28722 (*N*=125)
Gender (male/female)	157/131	122/110	308/250	–
Age (years, mean ± SD)	65.46 ± 13.26	64.73 ± 13.43	66.81 ± 13.32	65.33 ± 12.95
OS (years, mean ± SD)	2.61 ± 2.42	3.95 ± 2.56	–	5.39 ± 3.53
OS status (alive/dead)	219/69	139/93	–	55/70
DFS (years, mean ± SD)	203/62	3.65 ± 2.86	4.06 ± 3.37	4.98 ± 3.76
DFS status (0/1)	2.40 ± 2.31	145/55	380/177	92/33
Tumor stage (I/II/III/IV)	44/112/82/40	28/72/76/56	32/261/201/60	23/64/31/5

Abbreviations: DFS, disease-free survival; OS, overall survival; TCGA, The Cancer Genome Atlas.

### Feature transformation

**Figure 1:**
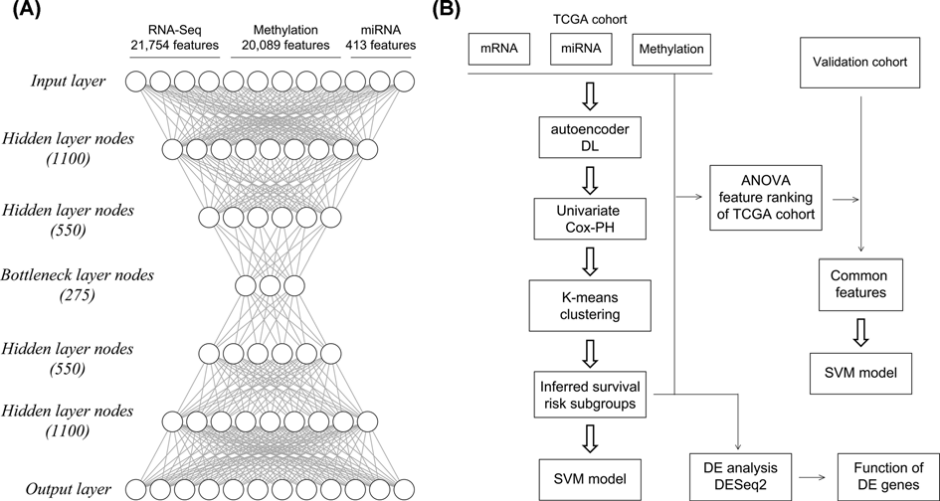
Overall study design (**A**) Autoencoder framework. (**B**) Construction and validation of the SVM model and further functional analysis.

We used multiomics data from TCGA set as the input for the autoencoder, a DL framework. As shown in Figure 1 [7], the three matrices were first unit norm-scaled by sample and then stacked to be a unique matrix. We applied *tanh* as the activation function for each layer. To train the autoencoder, we employed a gradient descent algorithm with 10 epochs and 50% dropout. With two hidden layers (550 and 1100 nodes, respectively), the autoencoder was implemented using the Python Keras library (https://github.com/fchollet/keras). By using the bottleneck layer of the autoencoder model, 275 transformed features were produced from the multiomics data.

It should be noted that: (1) this self-coding has no output noise setting, and the loss function is described below, and (2) this self-encoder is fully connected and self-coding. The detailed principle of self-coding is as follows:

Suppose *n*-dimensional features are input: *x* = (*x*_1_,.......,*x_n_*), and the purpose of self-coding is to reshape X′ by output X′ through a continuous hidden layer.

Given a layer, we used tanh as the activation function to connect the input X of each layer and the output y of each layer, which are shown as follows: $y=f_{i}(x)=\tanh(W_{i}\cdot xb_{i})$, where the sizes of X and Y are D and P, respectively, and *W*_i_ is the weight matrix of size *p* × *d*.

In the k layer, X′ is defined as: $x^{\prime }=F_{1\to k}(x)=f_{1}^{o}\cdots^{o}f_{k-1}^{o}f_{k}(x)$

$\;f_{k-1}^{o}f_{k}(x)=f_{k-1}(f_{k}(x))$ is the composite function of *f_k_*_-1_ and *f_k_*(*x*). To train self-coding, we aimed at different weight vectors *W*_i_._ We selected logloss as the objective function, which measures the error between input *x* and output *x*′:

$$\log loss(x,x^{\prime })=\sum_{k=1}^{d}x_{k}\log({x^{\prime }}_{k})(1-x_{k})\log(1-{x^{\prime }}_{k})$$

To prevent overfitting, we used the weight vector *W*_i_ plus L1 regularized penalty term *α_w_*, and in the active node, $F_{1\to k}(x)$ plus L2 regularization penalty term *α_a_*. Therefore, the objective function is defined as follows:

$$L(x,x^{\prime })=\log loss(x,x^{\prime })\sum_{i=1}^{k}\left(\alpha_{w}W_{i1}\alpha_{a}F_{1\to k}{\left(x\right)}_{2}^{2}\right)$$

### Univariable cox regression analysis of transformed features and *K*-means clustering

For each transformed feature, a univariable Cox proportional hazards (Cox-PH) model was constructed. The feature with a log-rank *P*-value <0.05 was considered significant. We clustered the samples of TCGA set using the *K*-means clustering algorithm in the nbclust package (https://cran.r-project.org/web/packages/NbClust/index.html) of R. Silhouette index [11], and the Calinski–Harabasz criterion [12] was used to select the optimal number of clusters. The nbcluster function of the R nbclust package was used to calculate the most clustered data when *k*-mer was 2-6. In fact, we selected the Silhouette index and Calinski–Harabasz criterion for the evaluation index. Finally, NbClust can obtain the highest clustering number according to the calculation results. The detailed values of the Silhouette index and Calinski–Harabasz criterion are shown in Supplementary Table S5. Following obtaining labels from *K*-means clustering, survival of different risk subgroups was compared using Kaplan–Meier survival curves and log-rank *t*-test. Log-rank *P*-value [13], C-index [14], and Brier score [15] were calculated to assess the accuracy of survival prediction in the identified risk subgroups.

### Comparative analysis of DL framework with principal component analysis, nonnegative matrix factorization, and *t*-distributed stochastic neighbor embedding

The DL framework was compared with other dimensionality reduction techniques, including principal component analysis (PCA) [16], nonnegative matrix factorization (NMF) [17], and *t*-distributed stochastic neighbor embedding (t-SNE) [18] for performance. For each method, 275 transformed features were used as features in the bottleneck layer of the DL framework. Using the same procedures mentioned above, the 275 transformed features underwent univariable Cox-PH model analysis, followed by *K*-means clustering of TCGA samples.

Moreover, the autoencoder based on three omics datasets was compared with the univariable Cox-PH model. Specifically, univariable Cox-PH analysis was conducted for all three omics datasets of TCGA set. The top 13 features were selected according to C-index score and were used to cluster the samples in TCGA set following the aforementioned *K*-means procedure (Figure 1).

### Data partitioning and robustness evaluation

Using the same cross-validation (CV)-like procedure described in a previous study [7], we randomly split the samples of TCGA dataset into five folds using the caret package of R, among which three folds were used as the training set and the other two folds were used as the test set. Consequently, 10 new combinations (folds) were acquired. For each new combination, the training set (60% of samples) was used to construct a model, which was then verified in the test set (40% samples). The robustness of the model was evaluated by calculating the log-rank *P*-value, C-index, and Brier score.

### Supervised classification

Following *K*-means clustering analysis, we performed analysis of variance (ANOVA) [19] with each omics dataset from TCGA. The top *N* features significantly associated with the labels of risk groups were identified based on ANOVA *F*-values. Default *N* values were set to 40 for RNA, 30 for methylation, and 30 for miRNA. The log-rank-*P* of this parameter was significant in both the training and validation sets, and the c-index was high.

The top 40 mRNAs and 30 methylation or 30 miRNA features identified by ANOVA were utilized to construct an SVM classifier, respectively, for predicting TCGA test data. The prediction accuracy of the SVM classification model was assessed using the log-rank *P*-value, C-index, and Brier score. The *penalize SVM* package of R was employed to carry out a grid search for the optimal combination of hyperparameters of the SVM model using five-fold CV and to develop SVM models.

### Confirmation using three independent validation sets

Three independent confirmation sets (E-GEOD-17538, E-GEOD-28722, and E-GEOD-39582), all of which contained RNA-seq data, were used for validation of the two survival risk subgroups. First, we selected common mRNA features between each validation set and TCGA set, respectively, which were further subjected to median scale normalization and robust scale normalization. After the two scaling steps, the corresponding top 40 mRNA features selected by ANOVA were identified to construct an SVM classifier.

### Bioinformatic analysis

Using TCGA data, we performed differential expression analysis in each individual omics layer between two survival risk groups identified by the autoencoder. The *DESeq2* package [20] (https://bioconductor.org/packages/release/bioc/html/DESeq2.html) of R was used to identify differentially expressed miRNAs and genes with |log_2_FC| >1 and FDR <0.05 as the selection cutoff. A moderate *t*-test using the *limma* package (https://bioconductor.org/packages/release/bioc/html/limma.html) in R was used to determine significant differences in methylation with |beta difference| >0.1 and FDR < 0.05 as the strict threshold.

In order to investigate whether DNA methylation affects gene expression, associations between methylation level and gene expression were evaluated by performing Pearson’s correlation analysis. A Pearson correlation coefficient < -0.5 and *P*-value <0.0001 were defined as significant differences. To study the regulatory relationships among differentially expressed mRNAs and miRNAs, potential target genes of the identified differentially expressed miRNAs were predicted using miRDB [21] (prediction score > 80) and TargetScan databases (probability of conserved targeting > 0.8, http://www.targetscan.org/vert_71/). Among the common target genes between the two databases, the differentially expressed genes (DEGs) between the two survival risk groups were selected to construct an miRNA–target gene network. Kyoto encyclopedia of genes and genomes (KEGG) pathway enrichment analysis of the identified DEGs was performed using the KOBAS tool. Pathways satisfying an FDR <0.05 were considered significant.

## Results

### Identification of two survival risk groups in TCGA multiomics data

**Figure 2:**
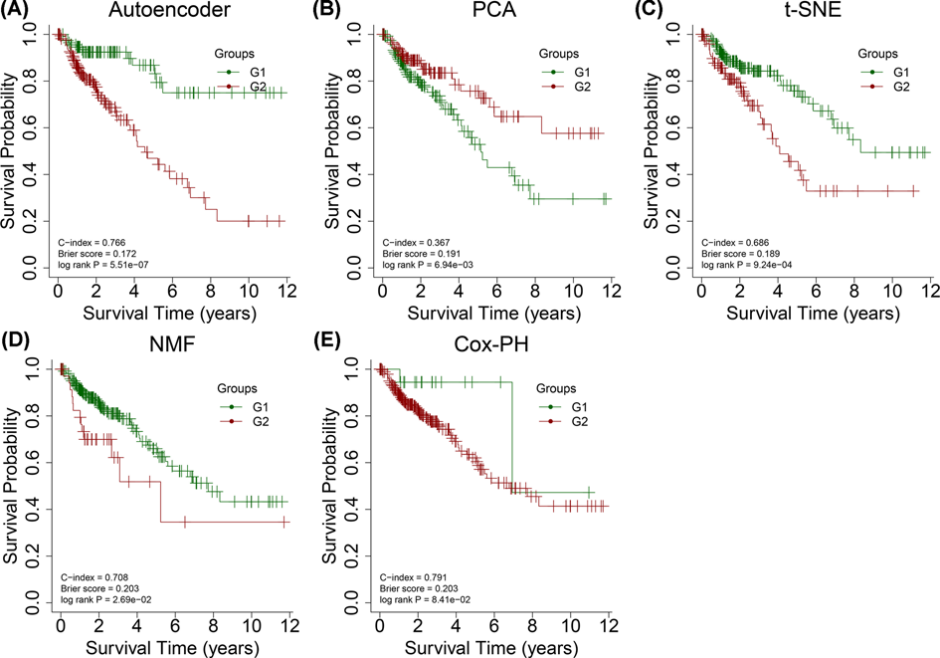
Kaplan–Meier (KM) curves for overall survival (OS) using different strategies KM curves for OS in The Cancer Genome Atlas (TCGA) set by using (**A**) autoencoder, (**B**) principal component analysis (PCA), (**C**) nonnegative matrix factorization (NMF), (**D**) t-distributed stochastic neighbor embedding (t-SNE), and (**E**) univariable Cox proportional hazards (PH) model.

The results shown here are in part based on data generated by TCGA Research Network: https://www.cancer.gov/tcga. After preprocessing TCGA multiomics data, we obtained 413 miRNAs from miRNA-seq, 21,754 genes from RNA-seq, and 20,089 genes from DNA methylation data as input features for the autoencoder. The three-omics data were stacked together and transformed into 275 new features by an autoencoder of two hidden layers (550 and 1100 nodes, respectively). Each of the 275 transformed features (detailed information in Supplementary Table S6) underwent a univariable Cox-PH regression model. The 13 features (detailed information in Supplementary Table S7) significantly associated with survival (log-rank *P*-value < 0.05) were then subjected to *K*-means clustering analysis. The optimal number of clusters was two. TCGA samples were classified into two survival risk groups (G1 and G2). As shown in Figure 2A, better survival was observed in the G1 group compared with the G2 group (log-rank *P*-value = 5.51e^−7^). The C-index and Brier scores were 0.766 and 0.172, respectively. These results suggested that this classification identified two different prognostic subtypes in patients with COAD.

We compared the autoencoder to other alternative methods, including PCA, NMF, t-SNE, and univariable Cox-PH model, for prognostic classification of COAD samples. Using each of the first three approaches, we obtained 275 transformed features, which is the same as the number of transformed features from the bottleneck layer of the autoencoder, and subjected these to a univariable Cox-PH model. In the Cox-PH approach, we performed univariable Cox-PH analysis on each input feature in the three omics data types. We ranked the features based on C-index values and selected the top 13 features, followed by *K*-means clustering. As depicted in Figure 2B–E, TCGA dataset was divided into two survival risk groups by each method. PCA (log-rank *P*-value = 0.191, C-index = 0.633, Brier score = 6.94e^−3^), NMF (log-rank *P*-value = 0.203, C-index = 0.708, Brier score = 2.69e^−2^), t-SNE (log-rank *P*-value = 0.189, C-index = 0.686, Brier score = 9.24e^−4^), and Cox-PH (log-rank *P*-value = 0.203, C-index = 0.791, Brier score = 8.41e^−2^) model failed to yield a significant log-rank *P*-value <0.05. We found that only the autoencoder could determine significant survival subgroups in patients with COAD.

To validate the robustness of the two inferred survival risk groups obtained by the autoencoder, a classification model was built using the SVM algorithm with CV (Figure 1B). TCGA samples were randomly separated into training (60%) and test (40%) sets. Table 2 shows a high C-index (0.73 ± 0.06), low Brier score (0.14 ± 0.01), and significant log-rank *P*-value (1.40e^−4^) for the three-omics training set on average. Similar results were observed for the three-omics test data (log-rank *P*-value = 2.92e^−2^, C-index = 0.64 ± 0.11, Brier score = 2.69e^−2^). With regard to the test of each omics dataset, this model also generated significant but marginally inferior results (Table 2). These results confirmed the robustness of the two inferential survival risk groups to the inherent stochastic processes of automatic encoder construction and training sample selection. Multiomics data proved to be superior to single-omics data for model construction.

**Table 2:** Performance assessment of the classification model using the CV procedure

Dataset	10-fold CV	C-index	Brier score	Log-rank *P*-value (geo.mean)
Training	3-omics training (60%)	0.73 ± 0.06	0.14 ± 0.01	1.40e^−4^
	RNA only	0.67 ± 0.07	0.15 ± 0.01	1.93e^−3^
	miRNA only	0.65 ± 0.05	0.14 ± 0.01	5.84e^−4^
	Methylation only	0.68 ± 0.09	0.15 ± 0.02	1.07e^−3^
Test	3-omics test (40%)	0.64 ± 0.11	0.16 ± 0.02	2.92e^−2^
	RNA only	0.63 ± 0.16	0.16 ± 0.02	4.07e^−2^
	miRNA only	0.62 ± 0.12	0.17 ± 0.02	3.96e^−2^
	Methylation only	0.60 ± 0.14	0.18 ± 0.02	4.72e^−2^

CV, cross-validation

### Survival risk subtypes were successfully validated in three independent validation datasets

To study the robustness of the classification model for predicting the prognosis of patients with COAD, we tested the model on three independent cohorts (E-GEOD-17538, E-GEOD-39582, and E-GEOD-28722). The numbers of common mRNAs shared by each validation set and TCGA set were 12959, 12959, and 12478, respectively. We selected the common top 40 features based on ANOVA F-value followed by SVM classification.

**Figure 3:**
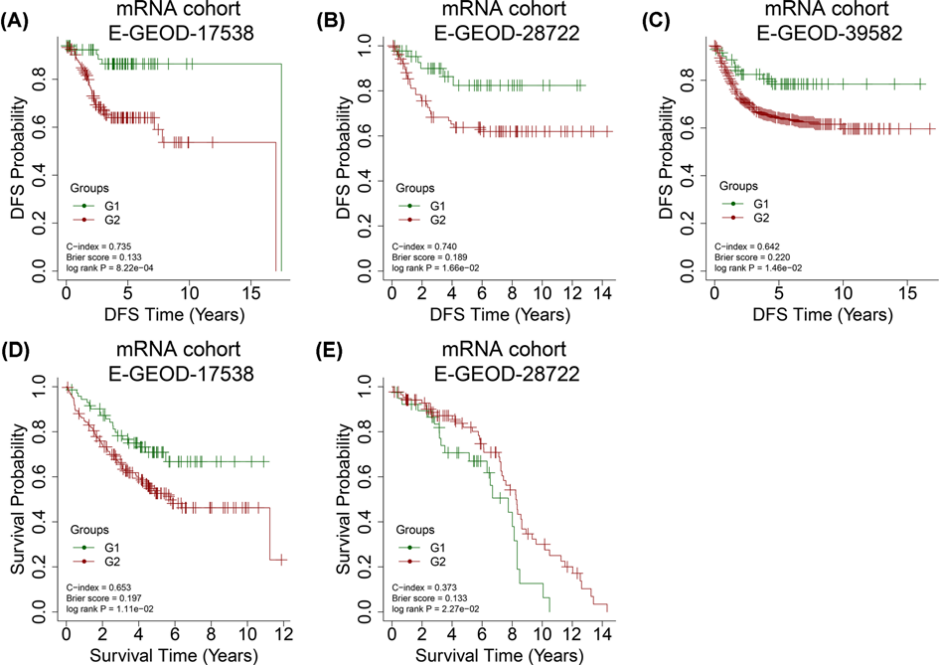
Kaplan-Meier (KM) curves for disease-free survival (DFS) and overall survival (OS) time in different datasets KM curves for DFS time in (**A**) E-GEOD-17538, (**B**) E-GEOD-39582, and (**C**) E-GEOD-28722 datasets and KM curves for OS time in (**D**) E-GEOD-17538 and (**E**) E-GEOD-28722 datasets using the survival classification model.

For E-GEOD-17538, we achieved a high C-index of 0.735, a low Brier score of 0.133, and a log-rank *P*-value of 8.22e^−4^ for disease-free survival (DFS) time (Figure 3A). The classification using overall survival (OS) time for E-GEOD-17538 generated the following values: log-rank *P*-value = 1.11e^−2^, C-index = 0.653, and Brier score = 0.197 (Figure 3D). Additionally, the classification generated good results for E-GEOD-28722 (log-rank *P*-value = 1.66e^−2^, C-index = 0.740, and Brier score = 0.189; Figure 3B) as well as E-GEOD-39582 (log-rank *P*-value = 1.46e^−2^, C-index = 0.642, and Brier score = 0.220; Figure 3C) datasets. These results proved the reliability of the two survival risk groups by autoencoders in COAD. The classification using OS time for E-GEOD-28722 generated the following values: log-rank *P*-value = 2.27e^−2^, C-index = 0.627, and Brier score = 0.133 (Figure 3E).

### Functional analysis of the two survival subgroups in TCGA dataset

**Figure 4:**
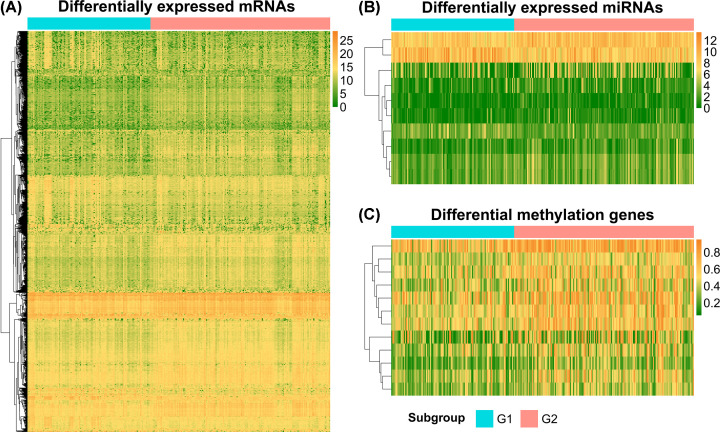
Heat maps for differentially expressed genes between two survival risk groups (**A**) mRNAs, (**B**) miRNAs, and (**C**) differentially methylated genes.

Between the two identified survival risk groups, we found 1271 DEGs, including 828 up-regulated and 443 down-regulated genes in the G2 group relative to the G1 group (|log_2_FC| > 1 and FDR < 0.05, Figure 4A). In total, 10 differentially expressed miRNAs (DEMs), consisting of 8 up-regulated and 2 down-regulated miRNAs (|log_2_FC| > 1 and FDR < 0.05), and 12 hypermethylated genes (FDR < 0.05 and |delta methylation| > 0.1) were found (Figure 4B,C).

**Figure 5:**
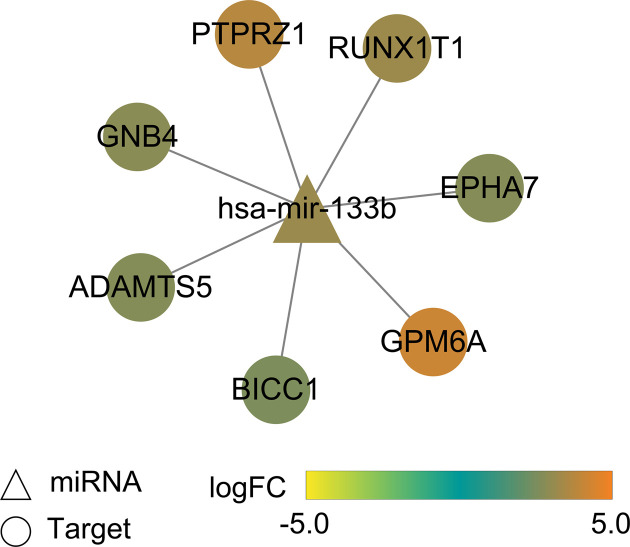
miR-133b-target gene network

Correlations of methylation β values with gene expression values were evaluated by calculating Pearson’s correlation coefficients. Expression levels of phospholipase A2 group IIA (*PLA2G2A*) and regenerating family member 4 (*REG4*) were significantly down-regulated by promoter hypermethylation (Pearson’s correlation coefficient < -0.5 and *P*-value < 0.001, Table 3). Potential target genes of 10 differentially expressed miRNAs were predicted using the miRDB (prediction score > 80) and TargetScan (Pct > 0.8) databases. The common target genes were mapped to DEGs. In total, seven target genes of mir-133b, including G protein subunit beta 4 (*GNB4*), protein tyrosine phosphatase receptor type Z1 (*PTPR Z1*), RUNX1 partner transcriptional co-repressor 1 (*RUNX1T1*), EPH receptor 7 (*EPHA7*), glycoprotein M6A (*GPM6A*), BicC family RNA binding protein 1 (*BICC1*), and ADAM metallopeptidase with thrombospondin type 1 motif 5 (*ADAMTS5*) were identified, and a miRNA target gene network was built (Figure 5).

**Table 3:** Correlation analysis of RNA expression and methylation data

Gene	RNA data FDR of RNA		Methylation data		Correlation coefficient	*p*-value
	Log_2_FC	FDR	Diff beta	FDR		
*PLA2G2A*	-1.26	1.14e^−5^	0.11	2.46e^−7^	-0.53	3.26e^−22^
*REG4*	-1.28	6.64e^−3^	0.10	1.33e^−5^	-0.61	3.13e^−30^

**Figure 6:**
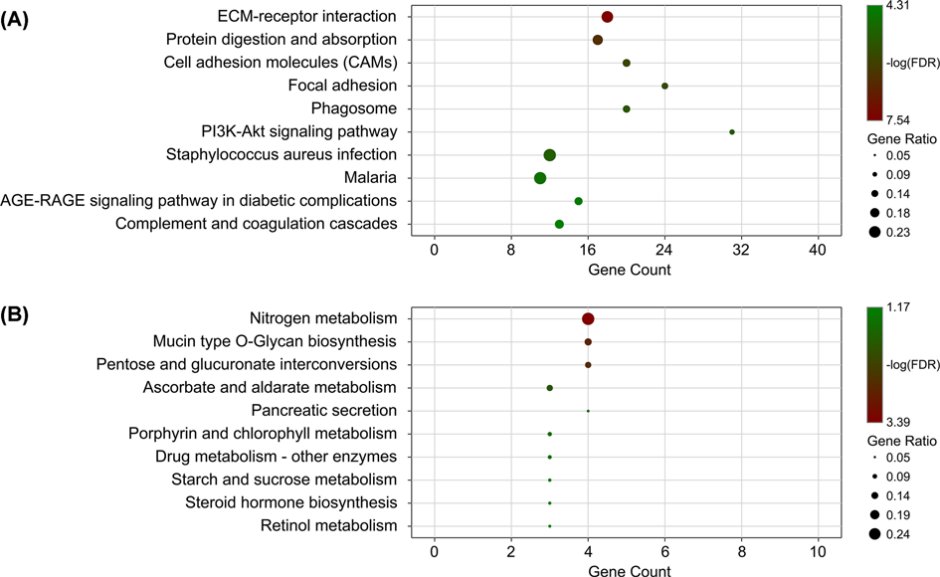
Significantly enriched pathways for differentially expressed genes (**A**) top10 significant pathways for up-regulated genes; (**B**) top 10 significant pathways for down-regulated genes.

We performed pathway enrichment analysis for DEGs. The up-regulated genes were significantly associated with ECM–receptor interaction, focal adhesion, and PI_3_K-Akt signaling pathways (Figure 6A, FDR < 0.05), while the down-regulated genes were significantly associated with nitrogen metabolism, mucin type O-glycan biosynthesis, and pentose and glucuronate interconversions (Figure 6B, FDR < 0.05).

## Discussion

At present, the survival and prognosis of patients with COAD are poor. Accurate stratification of patients with COAD indicative of prognosis would help to select the optimal therapy for each patient. In the present study, RNA-Seq, miRNA-Seq, and DNA methylation data of the same patients were downloaded from TCGA to develop a survival prediction model using the DL framework. The present model was based only on TCGA database and now the GEO database. Except for TCGA database, no other databases such as GEO database contained cancer samples with ‘RNA-seq’, ‘miRNA’, and ‘methylation’ data; thus, the GEO database could not be used to verify the dimension reduction of the autoencoder model. Two risk subgroups with robust survival differences were inferred by the autoencoder framework based on multiomics data. According to the C-index value, log-rank *P*-value, and Brier score, the autoencoder algorithm was preferred for selecting survival-related features to other alternative approaches, including PCA, NMF, t-SNE, and the univariable Cox-PH model, emphasizing the utility of this approach. The reliability of the two inferred risk groups was confirmed using the CV procedure. Moreover, this classification model generated good results in terms of C-index, Log-rank *P*-value, and Brier score on three additional validation datasets containing RNA-seq data, confirming its predictive efficiency. In the present study, the autoencoder model was used to reduce the dimension of multiomics data. The bottleneck feature was the dimension reduction feature of the autoencoder model, which was combined with the traditional risk grouping method. Our emphasis was on the advantages of multiomics integration, and we also compared the autoencoder with other dimensionality reduction methods. We aimed to obtain transformation characteristics by multiomics integration dimension reduction using DL and carried out risk assessment according to the dimensionality reduction features. Our results indicated that the present dimensionality reduction method was superior to other dimensionality reduction methods as well as uniomics research, which could avoid differences in data platforms and omics of multiomics integration. The present study will help improve the prognosis of patients with COAD. The three confirmation cohorts used in the present study consisted of RNA-seq data only. Large cohort studies with good quality samples are anticipated to further validate the predictive utility of this two-risk group-specific model. We performed integrative bioinformatics analysis to search for critical molecules involved in the biology of COAD. A total of 1271 DEGs, 10 DEMs, and 12 hypermethylated genes were identified between the G1 and G2 risk subgroups. Notably, down-regulation of *PLA2G2A* and *REG4* by promoter hypermethylation was observed. Phospholipase A2 is an enzyme related to the hydrolysis of fatty acyl ester. Expression of *PLA2G2A* and *REG4* is of prognostic value for patients with stage II CRC [22,23]. Furthermore, miR-133b and its seven target genes (*GNB4, PTPRZ1, RUNX1T1, EPHA7, GPM6A, BICC1*, and *ADAMTS5*) were differentially expressed between the two risk groups. All the eight genes are associated with cell proliferation, invasion, and migration enhancement and poor prognosis in various cancers [24]. miR-133b expression is decreased in CRC and suppresses CRC metastasis, which is associated with the OS of CRC [25,26]. miR-133b down-regulation promotes CRC invasion and migration by modulating *CXCR4* [24]. *GNB4*, a signal transduction molecule involved in the PI_3_K-AKT pathway, is hypermethylated and down-regulated in both CRC cell lines and colon cancers [27]. PTPRs are a subgroup of tyrosine phosphatases that participate in regulating the cell signaling events of several critical biological processes, such as proliferation, apoptosis, and migration [28]. *PTPRZ1* expression is elevated in CRC, implicating its involvement in CRC development [29]. RUNX1T1, a transcriptional co-repressor, acts as a critical regulator of leukemogenesis. *RUNXIT1* may play a suppressive role in CRC progression [30]. The involvement of Eph/ephrin signaling in a wide range of biological processes related to tumor progression and metastasis, such as cell attachment, migration, and angiogenesis, has been characterized [31]. The down-regulation of *EphA7* by hypermethylation occurs in CRC [32]. GPM6A is a transmembrane protein that plays an important role in the differentiation and neuronal migration of neurons [33]. Overexpression of miR-133b in neuronal cultures leads to the downregulation of *GPM6A*, suggesting that *GPM6A* is a novel target for epigenetic regulation during prenatal stress [34]. The gene product Bicc1 is an RNA-binding molecule involved in regulating various proteins at the post-transcriptional level [35]. *BICC* is a genetic determinant of osteoblastogenesis and bone mineral density [36]. ADAMTS5 is a secreted proteinase that participates in cell adhesion, proliferation, and migration. *ADAMTS5* is overexpressed in CRC, promoting CRC metastasis and cancer cell invasion [37]. High expression of *ADAMTS5* is a potent biomarker for lymphatic invasion and lymph node metastasis in CRC [38]. These results indicate that these molecules may be used as promising biomarkers and therapeutic targets for COAD.

Functional analysis of the up-regulated DEGs revealed significant enrichment of various signaling pathways, such as ECM–receptor interaction, focal adhesion, and the PI3K-Akt signaling pathway. The down-regulated genes were significantly involved in several signaling pathways associated with glucose metabolism. A rich body of evidence has shown that the PI_3_K-Akt signaling pathway plays an essential role in the progression of colon cancer and is a promising target for cancer treatment [39,40]. Glucose intake is high in cancer cells together with the production of lactic acid [41]. However, these results were found based on bioinformatic analysis. We hope that the results of the present study will be beneficial for elucidating the possible pathogenesis of COAD.

Here, we performed an extensive study based on published data and bioinformatic analysis. The results of the present study should be further validated using *in vitro* or *in vivo* models. We hope that the results of this study will be beneficial to future research.

## Conclusion

The present study robustly distinguishes survival subpopulations of patients with COAD using DL-based multiomics integration. This classification is of direct clinical importance and contributes to improved outcomes in patients with COAD. miR-133b, *GNB4, PTPRZ1, RUNX1T1, EPHA7, GPM6A, BICC1*, and *ADAMTS5* may be important molecular targets for COAD.

## Supplementary Material

Supplementary Tables S1-S7

## Data Availability

All data used and/or analyzed in this study are available from the TCGA database (https://gdc-portal.nci.nih.gov/) or the EBI Array database (https://www.ebi.ac.uk/arrayexpress/).

## Abbreviations

COAD: colon adenocarcinoma
DFS: disease-free survival
DL: deep learning
NMF: nonnegative matrix factorization
OS: overall survival
PCA: principal component analysis
TCGA: The Cancer Genome Atlas
t-SNE: *t*-distributed stochastic neighbor embedding
